# Emerging Insights into the Applicability of Essential Oils in the Management of Acne Vulgaris

**DOI:** 10.3390/molecules28176395

**Published:** 2023-09-01

**Authors:** Alexa Florina Bungau, Andrei-Flavius Radu, Simona Gabriela Bungau, Cosmin Mihai Vesa, Delia Mirela Tit, Anamaria Lavinia Purza, Laura Maria Endres

**Affiliations:** 1 Doctoral School of Biological and Biomedical Sciences, University of Oradea, 410087 Oradea, Romania; pradaalexaflorina@gmail.com (A.F.B.); v_cosmin_15@yahoo.com (C.M.V.); mirela_tit@yahoo.com (D.M.T.); 2 Department of Preclinical Disciplines, Faculty of Medicine and Pharmacy, University of Oradea, 410073 Oradea, Romania; 3 Department of Pharmacy, Faculty of Medicine and Pharmacy, University of Oradea, 410028 Oradea, Romania; purza_lavinia@yahoo.com; 4 Department of Psycho-Neurosciences and Recovery, Faculty of Medicine and Pharmacy, University of Oradea, 410073 Oradea, Romania; laura_endres@yahoo.com

**Keywords:** acne vulgaris, antioxidants, essential oils, phytochemistry

## Abstract

The occurrence of pustules, comedones, nodules, and cysts defines acne vulgaris, a prevalent chronic inflammatory dermatological condition. In the past few decades, essential oils extracted from varied natural sources have acquired recognition due to their potential medicinal applications in acne therapy. However, there is not yet sufficient medical data to fully characterize this interaction. Multiple factors contribute to the development of acne vulgaris, including excessive sebaceous production, inflammatory processes, hyperkeratinization, and infection with *Cutibacterium acnes*. Essential oils, including oregano, lavender, lemon grass, myrtle, lemon, thyme, eucalyptus, rosemary, and tea tree, have been found to possess anti-inflammatory, antioxidant, and antimicrobial properties, which may target the multifactorial causes of acne. Analytical methods for determining antioxidant potential (i.e., total phenolic content, diphenyl-1-picrylhydrazyl free radical scavenging assay, reducing power assay, ferrous ion chelating activity, thiobarbituric acid reactive species assay, β-carotene bleaching assay, etc.) are essential for the evaluation of these essential oils, and their method optimization is crucial. Further studies could include the development of novel acne treatments incorporating essential oils and an assessment of their efficacy in large clinical trials. In addition, further research is necessary to ascertain the mechanisms of action of essential oils and their optimal doses and safety profiles for optimal implementation in the management of acne vulgaris.

## 1. Introduction

Acne vulgaris is a prevalent, persistent, inflammatory disorder of the pilosebaceous complex that is mainly triggered by elevated production of sebum, hyperkeratinization of the hair follicle, bacteria overgrowth, and inflammatory conditions. Furthermore, the condition is marked by the persistent or recurrent occurrence of comedones, pustules, and erythematous papules on the skin of the face, upper limbs, and neck [1,2]. The onset of acne vulgaris has a significant impact on the quality of life of an individual, influencing both confidence and social and psychological growth. Physicians are confronted with numerous over-the-counter or prescribed acne therapies, rendering it challenging to select the one which is most efficient and safe [3].

Regarded as essential to the conventional causes of acne vulgaris are elevated sebum production, endocrinological components including androgens, aberrant keratinization of the follicles, consequent inflammation, and excessive growth and proliferation of *Cutibacterium acnes* [4]. Acne can range from a moderate form to aggressive nodulocystic acne, which is highly inflammatory and affects the back, chest, and face. According to current research, *Cutibacterium acnes*, a type of anaerobic microorganism found in the sebum that is accumulated in follicular ducts, proliferates as a result of a higher activity level of the sebaceous glands caused by androgens [5]. Moreover, there is confirmation that distinct inflammatory cascades emerge early, throughout the initial subclinical development and evident appearance of acne vulgaris lesions, subsequently, during advancement, and ultimately, during resolution, which includes scarring. Additionally, it has been demonstrated that subclinical inflammatory processes occur prior to or simultaneously with microcomedone development [6].

Androgens, including testosterone and its derivative dihydrotestosterone, have an essential function in the onset of acne [7]. Both women and men produce these hormones, but men have significantly higher concentrations. Androgens may trigger acne by stimulating the sebaceous glands, which produce sebum. Elevated androgen levels cause excessive production of sebum, which obstructs hair follicles and results in the emergence of comedones [8]. In addition, sebum facilitates the rapid growth of *Cutibacteirum acnes*, a bacterium that contributes further to the occurrence of acne lesions [9,10]. Hormonal imbalances, menstruation, and puberty can all exacerbate acne. Androgen levels increase during puberty, causing an increase in sebum secretion and the development of acne. Similarly, hormonal fluctuations during the menstrual cycle, specifically the rise in androgen levels prior to menstruation, can induce eruptions of acne in specific individuals [11]. Knowledge of the function of sex hormones in acne vulgaris has resulted in the design of hormonal therapies for the treatment of acne. Such therapies, including anti-androgens and oral contraceptives, seek to decrease sebum production and regulate hormone amounts [7].

The therapeutic management of acne vulgaris is determined by its degree of severity. Patients with widespread papules and pustules indicating severe acne typically have both topical and oral therapies administered. Acne vulgaris may be treated with retinoids, salicylic acids, benzoyl peroxide, azelaic acids, isotretinoids, and alpha-hydroxy acids, as well as hormone-related, anti-androgen, or antiseborrheic medications. After a period of between six and eight weeks, the effectiveness, adherence, and side effects are evaluated, and the treatment plan is modified accordingly [12,13,14].

Herbal remedies have a lengthy history of usage and have been demonstrated to have minimal adverse effects [15]. Aside from common illnesses such as colds and infections, medicinal plants have been beneficial in the prevention and treatment of a wide range of complex ailments, including malignancies, hypertension, diabetes, cardiovascular diseases, and even skin disorders [16,17].

In the majority of medical contexts, a combination of strategies appears to be the optimal treatment for acne, offering the most beneficial outcomes. Herbal anti-acne remedies consist of different plant extracts, combinations of plant extracts, and phytochemicals with topical (e.g., lotions, creams, gels) and oral (e.g., capsules and tablets) administration [18]. Among these herbal medicines, an important role is played by essential oils, which are included within cleansers, mechanical treatments, vitamins, and leave-on products in the category of over-the-counter acne therapies [19].

Essential oils, along with their volatile constituents, possess the ability to permeate the skin and facilitate the enhanced penetration of active compounds into deeper skin layers. These effects are achieved through the disruption of the structured lipid arrangement between corneocytes in the stratum corneum and interaction with the intercellular domain of proteins, inducing alterations in their conformation [20]. These mechanisms mean that topical administration of essential oils (e.g., lotions, face wash, gel, creams) allows phytocompounds to reach the hair follicles region for direct action on *Cutibacterium acnes* [18].

Essential oils are intricate mixtures of volatile phytochemical extracts characterized by distinct olfactory characteristics. These extracts, obtained from diverse plant parts, encompass an array of natural volatile organic compounds. The diversity of sources containing volatile oils can be observed by the fact that leaves serve as the primary source for obtaining eucalyptus and peppermint oils (e.g., eucalyptol and menthol), while flowers contribute to the extraction of lavender and rose oils (e.g., linalool and citronellol). Moreover, roots yield calamus oils, while sap is the origin of benzoin and frankincense oils. These oils represent intricate synergies of constituents, variably contributing to therapeutic attributes predicated on their relative ratios [21].

Antimicrobial activity is linked to essential oils that are high in acids, phenols, aromatic aldehydes, phenyl methyl esters, and in alcohols and terpenes with a carbon chain length of 10 atoms. This property can be attributed to the interplay of these bioactive constituents with microbial cell membranes and enzymatic processes, underscoring their potential in pharmacological and clinical applications [22].

**Figure 1:**
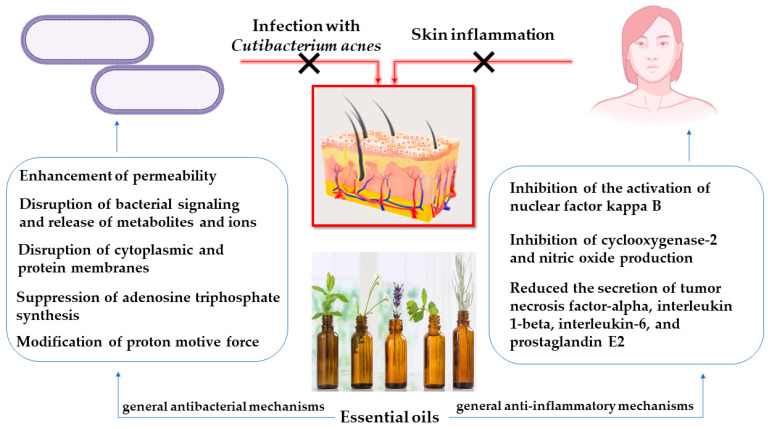
General antibacterial and anti-inflammatory mechanisms of essential oils in acne vulgaris.

The most relevant effects of the compounds in the essential oils for the management of acne vulgaris are antibacterial, targeting *Cutibacterium acnes,* and anti-inflammatory, controlling inflammation secondary to infection. The general antibacterial and anti-inflammatory mechanisms of essential oils in acne vulgaris [23] are illustrated in Figure 1.

The antibacterial activity of essential oils is attributed to their constituents’ ability to interact with cell membranes, disrupting microbial integrity and leading to cell death. However, the bioactive components of essential oils can target multiple cellular sites, primarily involving cytoplasmic coagulation, inhibition of ATP-producing enzymes, modulation of ion transport, cell wall disruption, and bacterial membrane disintegration. In terms of anti-inflammatory effects, essential oils showed activities such as inhibiting the activation of nuclear factor-kappa B induced by *Cutibacterium acnes* [24,25]. They also reduced nitric oxide (NO) and cyclooxygenase-2 production, mitigated the secretion of tumor necrosis factor-alpha (TNF-α) and interleukin-8 (IL-8) in THP-1 cells stimulated by *Cutibacterium acnes*, and demonstrated anti-inflammatory properties by decreasing the secretion of TNF-α, IL-1β, IL-6, NO, and prostaglandin E2 in RAW 264.7 cells, particularly when exposed to lipopolysaccharide (LPS) stimulation [23].

Anti-acne remedies include antiseptic essential oils (e.g., lemon oil, petigrain oil, or tea tree oil) along with bactericidal and anti-inflammatory oils that avoid clogging of the skin’s sebum-producing glands (e.g., thyme, lavender, basil, or bergamot oil) [26]. Numerous experimental studies have been conducted to test the antimicrobial effect of essential oils against *Cutibacterium acnes* in order to establish an optimal dose, a suitable safety profile, and to classify them according to antimicrobial potency [26,27,28]. Furthermore, clinical studies, although less numerous than in vitro studies, have shown promising results for the use of essential oils in the management of acne vulgaris [29], showing superiority in some formulations over benzoyl peroxide [30] or clindamycin [31].

Research into the properties of free radicals and antioxidants results in a medical revolution that includes promising new approaches to disease management. Research into effective, harmless natural compounds with antioxidant activity has increased over the past decade [32]. Moreover, alongside endogenous protective antioxidant systems, plant-derived antioxidants in the form of essential oils can serve as an adequate alternative for the management of inflammatory-related acne vulgaris [21].

The purpose of the present narrative review is to highlight the potential role of essential oils in the therapeutic management of acne vulgaris in the context of the fact that the applicability of essential oils as adjuvant therapies for different types of patients with acne vulgaris is not yet fully characterized. This paper presents an overview of the pathogenesis of acne vulgaris and addresses the antioxidant, antimicrobial, and anti-inflammatory properties of essential oils that render them beneficial for the management of acne vulgaris. Furthermore, the analytical methods utilized for assessing the antioxidant potential of essential oils have been described, which can help researchers devise and conduct future research.

## 2. Pathophysiology of Acne Vulgaris

*Cutibacterium acnes* generates chemotactic molecules for macrophages and triggers the discharge of hydrolytic enzymes related to follicular wall injury, thereby initiating an inflammatory response [33]. Furthermore, it has been observed that reactive oxygen species (ROS) released by neutrophils throughout the follicular wall in response to pathogen action trigger the onset of inflammation and play an integral role in the development of the illness [34].

MicroRNAs (miRNAs) are short RNA molecules composed of 19–25 nucleotides that operate as post-transcriptional regulators of gene expression. They exhibit varying levels of expression in diverse pathological contexts, including skin conditions. This variability in expression patterns has enabled the characterization of distinctive miRNA ‘signature’ profiles corresponding to different disease conditions [35]. Patients with moderate to severe acne had statistically considerably greater amounts of miRNA-21 in their plasma when compared to the patients in the control group [36]. Moreover, plasma levels of miRNA-31 and miRNA-200a were slightly higher in patients with severe acne versus those in the control group. However, the difference was not considered statistically significant. The detection of increased concentrations of these particular miRNAs in the serum of individuals afflicted by acne presents the prospect of utilizing a blood test for the identification of individuals susceptible to scarring. This approach could enable early intervention with appropriate therapeutic measures [35]. Serum malondialdehyde (MDA) levels were increased in individuals with severe acne in comparison with the control group, while serum concentrations of glutathione (GSH) were reduced. These findings indicate that oxidative stress is implicated in the etiopathogenesis of acne vulgaris and that miRNA-21 may constitute an essential part in the course of the development of acne vulgaris [36].

In addition, elevated thiobarbituric acid reactive substances (TBARS) values were indicative of an enhanced oxidative damage burden in acne patients [37]. MDA, a reactive species acknowledged as a marker of heightened oxidative stress, has been reported to be higher in various research studies [38,39]. Recent study evidence indicates that acne is characterized by reduced blood superoxide dismutase (SOD), glutathione peroxidase (GSH-Px), and elevated MDA levels compared to healthy controls [37].

In the past few decades, it has been indicated conclusively that the onset of acne is due to a mixture of predisposing genetic factors and triggers from the environment, with the follicular invasion by *Cutibacterium acnes* assuming a key role. *Cutibacterium acnes* exhibits multiple processes that may contribute to the occurrence of acne inflammation of the skin, including the onset of sebogenesis, facilitation of the development of follicular hyperkeratinization, triggering of a response of inflammation by a release of proinflammatory compounds, and induction of innate immunity, which is then followed by a *Cutibacterium acnes*-specific adaptive immune system response [40].

Ozlu et al. detected Toll-like receptor (TLR) 2 macrophages in lesions associated with acne and in surrounding areas of pilosebaceous units, then demonstrated that the number of these cells multiplied as the disorder advanced [41]. In addition, Jugeau et al. reported an upregulation of both TLR4 and TLR2 expression in keratinocytes of patients with acne associated with inflammation. Therefore, it has been hypothesized that *Cutibacterium acnes* stimulates cells during inflammatory acne by interacting with TLR2 and TLR4 [42]. The epidermis contains numerous varieties of TLR-expressing cells, which include keratinocytes and Langerhans cell types [43].

**Figure 2:**
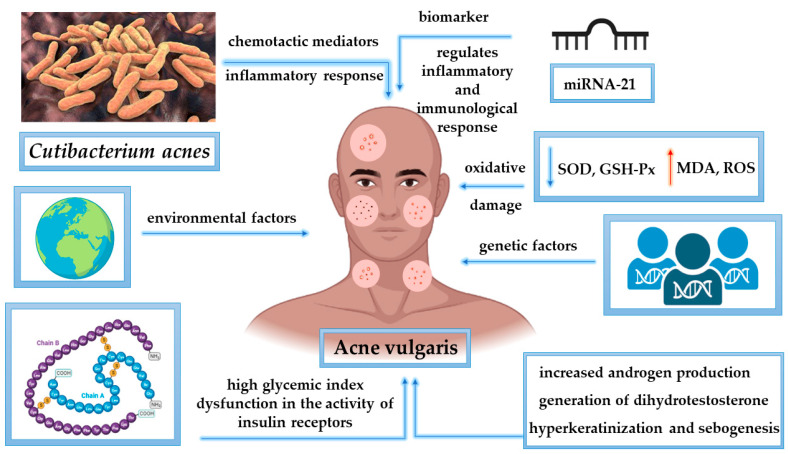
Pathophysiological mechanisms involved in acne vulgaris development. miRNA-21, microRNA 21; SOD, superoxide dismutase; GSH-Px, glutathione peroxidase; MDA, malondialdehyde; ROS, reactive oxygen species.

Serological evidence supports the significance of the mammalian target of rapamycin complex 1 (mTORC1) signaling pathway in the interaction between insulin, androgens, insulin-like growth factor (IGF1), and a high-glycemic-index diet in acne [44,45]. Peroxisome proliferator-activated receptors (PPARs) are involved in differentiation as well as anti-inflammatory mechanisms. Poorly differentiated sebocytes exhibited decreased expression of PPAR and elevated levels of IGF-1 receptors and insulin, leading to the generation of sebum and mediators of inflammation resembling acne [46]. Figure 2 shows the most important processes involved in the onset and development of acne vulgaris.

## 3. Anti-Inflammatory and Anti-Microbial Activity of Certain Essential Oils

### 3.1. Oregano Essential Oil

The examined microorganisms that cause acne were effectively neutralized by oregano essential oil. Oregano, thymol, and thyme were the most effective three antimicrobial agents on *Cutibacterium acnes* when minimum inhibitory concentration, disc diffusion, and minimal bactericidal concentration assays were used. Regarding *Cutibacterium acnes* and *Staphylococcus epidermidis*, lemon grass and tea tree essential oils showed moderate antibacterial activity. Chamomile, menthe, and lavender essential oils were ineffective against either of the studied acne-causing microorganisms [47].

Carvacrol, also known as 2-methyl-5-(1-methylethyl)-phenol, and thymol, also known as 2-isopropyl-5-methylphenol, are the isomeric phenolic monoterpenes that, based on the available information, are the representative substances that exert the major therapeutic effects discovered in various *Origanum vulgare* essential oils [48].

These compounds are biosynthesized through p-cymene hydroxylation and aromatization. Nevertheless, *Origanum vulgare* essential oil has a complex chemical composition that includes several distinct chemicals, like tannins, acyclic, monocyclic and bicyclic monoterpenes (i.e., terpinen-4-ol, γ-terpinene, *trans*-sabinene, *p*-cymene, linalool, geraniol, α-pinene and β -myrcene), sesquiterpenes (i.e., β-caryophyllene, germacrene-D, 1,8-cineole β-citronellol), phenolic acids (i.e., chlorogenic and rosmarinic acids), and flavonoids (i.e., apigenin, luteolin, and quercetin) [49].

After the extraction of oregano leaves with ethanol, four components, determined with high-performance liquid chromatography, were shown to have significant antioxidant activity. These substances included apigenin, rosmarinic acid, carvacrol, and quercetin. Results showed that ethanol oregano extracts effectively reduced *Cutibacterium acnes*-induced skin inflammation, as shown by biopsy weight (37%) and ear thickness (32%), using the mouse ear edema model. In another research investigation, ethanol oregano extracts decreased the synthesis of IL-1β, IL-8, and TNF-α up to 37%, 40%, and 18%, respectively, in addition to reducing the expression of the mentioned pro-inflammatory mediators at the transcriptional stage, utilizing the co-culture of *Cutibacterium acnes* and human THP-1 monocytes [50].

After treatment with oregano essential oil, Cheng et al. evaluated the anti-inflammatory activity on LPS-treated murine macrophage cells. Following a 12-h treatment with oregano essential oil (2.5, 5, or 10 μg/mL), the results of the analysis with the enzyme-linked immunosorbent assay commercial kits indicated lower levels of IL-1, IL-6, and TNF-α [51].

### 3.2. Cymbopogon Martinii Essential Oil

Due to its antibacterial qualities, *Cymbopogon martinii*, commonly referred to as palmarosa or lemon grass, has been utilized in aromatherapy as a skin tonic. Furthermore, it has been utilized in Ayurvedic medicine to treat skin issues and ease nerve discomfort. In an in vitro study, the immunomodulatory effects of the essential oil of *Cymbopogon martinii* and its constituent geraniol were assessed in terms of their impact on the production of pro-inflammatory (e.g., TNF-α) and anti-inflammatory (e.g., IL-10) cytokines by human monocytes. The presence of *Cymbopogon martinii* essential oil did not exhibit any discernible influence on TNF-α production. However, across various concentrations of *Cymbopogon martinii* essential oil, there was a notable increase in the production of IL-10 by human monocytes [52].

In an experimental study, the essential oil of *Cymbopogon martinii* was examined as a possible topical treatment for acne vulgaris. Gas chromatography coupled with mass spectrometry (GC-MS) profiling was carried out, and *Cutibacterium acnes* was used as a microorganism to test the antimicrobial potential. The main phytochemical detected in the oil was geraniol, and topical use of the oil was proven to be safe. The levels for the lowest bactericidal and inhibitory concentrations were 16 μL/mL. TNF-α, IL-8 and IL-12 cytokine concentrations were decreased by essential oil, and tyrosinase was suppressed [53].

### 3.3. Lavender Essential Oil

Lavender essential oil, which is obtained by processing the flowering tops of *Lavandula angustifolia* Mill. *(Lamiaceae*), emerges as a potential candidate for a natural product that can boost the synergistic action of some antiseptics, like over-the-counter medications. The pharmaceutical sector uses lavender essential oil in a variety of products while the cosmetics industry uses it as a scent component [54].

The main goal of an experimental study was to assess how *Lavandula angustifolia* Mill. Essential oil (LEO) influences the acute response of the body to inflammation. Nuclear magnetic resonance spectroscopy and GC-MS techniques were used to examine LEO and revealed that camphor (22.12%), borneol (22.63%), and 1,8-cineole (39.83%) predominated. In vitro cytotoxicity was not observed for LEO at doses of 0.5, 1, 3, and 10 μg/mL. Furthermore, in vitro leukocyte chemotaxis was not induced by LEO. In the croton oil-induced ear edema model, applying LEO topically, at dosages of 0.25, 0.5, and 1 mg/ear decreased the development of edema, nitric oxide generation, and myeloperoxidase activity [55].

In an in vivo study, transcriptional analysis was used to examine the way a treatment with LEO affects the macrophages’ reaction to *Staphylococcus aureus* infection. According to the findings, the therapy enhances phagocytic activity and encourages the macrophages control of bacterial multiplication inside cells. The obtained results demonstrated that these stimuli are accompanied by the expression of the CYBB and NCF4 genes, which are linked to the generation of ROS. By suppressing the main pro-inflammatory cytokines and their associated receptors and promoting the transcription of the heme oxygenase-1 gene, the essential oil treatment also counteracted the inflammatory signals caused by *Staphylococcus aureus* [56].

### 3.4. Myrtle Essential Oil

In Persian medicine, myrtle (*Myrtus communis*) has been traditionally recommended for diverse skin conditions, both topically and internally. Various in vitro and in vivo investigations on myrtle have revealed that it possesses antiproliferative, antimicrobial, and anti-inflammatory activities [57].

The leaves of the myrtle plant contain two distinct nonprenylated, oligomeric acylphloroglucinols called semi-myrtucommulone (S-MC) and myrtucommulone (MC). By suppressing cyclooxygenase-1 and 5-lipoxygenase in vivo and in vitro with an IC50 concentration in the interval between 1.8 and 29 µM, it has been demonstrated in the study for the first time that MC and S-MC effectively decrease the production of eicosanoids [58].

In the myrtle leaves, reduced levels of flavonoids (i.e., quercetin 3-O-rhamnoside and quercetin 3-O-galactoside) and phenolic acids (i.e., ellagic, caffeic, and gallic acids) were also found. Furthermore, significant amounts of flavonoids, like myricetin and galloyl derivatives of catechin, have been identified. From the leaves, it was possible to separate the polyphenolic substances, hydrolysable tannins, and myricetin glycosides. The main terpenoids and their derivatives, including α-terpineol, caryophyllene oxide, α-pinene, 1,8-cineole, geranyl butyrate, linalool, geraniol, and geranyl acetate, were also identified in the oil sample from myrtle leaves [59].

The antioxidant action of the studied extracts was influenced by several variables, including extraction type, extraction solvent, storage, and the plant component used. Myrtle leaf extracts were found to have anti-inflammatory properties in a variety of models. The flavonoids and hydrolysable tannins were assumed to have this characteristic, although nonprenylated acylphloroglucinols, such as S-MC and MC, have also demonstrated a noteworthy function in that action. Myrtle extracts may be used to stabilize challenging lipid systems, as prebiotics in food products, and as a potential therapy for the treatment of inflammations, based on the biological activities that have been identified until now [60].

### 3.5. Lemon and Other Citrus Essential Oil

Lemon, one of the most therapeutic fruits in the world, is a naturally acidic fruit that can be consumed by humans. It is an important source of vitamin C, vitamin B1, iron, phosphorus, calcium, vitamin B2, and other nutrients. Lemon has many health-enhancing properties that are good for the human body, including preventing and eradicating skin pigmentation, enhancing vision and adaptation to the dark, reducing weariness, and many others [61].

Numerous scientific investigations have been conducted on *Citrus limon* fruit extracts, essential oil, and the active substances obtained from these raw materials in an effort to support the feasibility of their application in cosmetics. To treat acne and smooth the skin on the face, *Citrus limon* fruit juice is combined with honey, egg albumin, and cucumber in Tanzania [62].

A minimal inhibitory concentration (MIC) of 20 mL/L of limonene was demonstrated by Han et al. to be efficient in preventing the development of *Staphylococcus aureus* ATCC 6538. It was found that limonene damaged cell membranes, increased membrane permeability, and decreased respiratory system metabolic action while also destroying cell shape and the integrity of bacterial cell walls. There are several potential applications for *Citrus limon* essential oil and limonene as acne treatments due to their antibacterial properties [63].

### 3.6. Eucalyptus Essential Oil

The usage of *Eucalyptus* essential oil and its primary component, eucalyptol, in the food, cosmetic, and pharmaceutical sectors is considerable, and its clinical application as an adjuvant in the treatment of infectious and inflammatory illnesses has lately spread throughout the world [64].

A variety of microorganisms, such as viruses, methicillin-resistant *Staphylococcus aureus*, *Mycobacterium tuberculosis*, and fungi, including *Candida* species, are susceptible to the antimicrobial effects of eucalyptus oil and its main component, 1,8-cineole. It has been observed that this essential oil also possesses immunostimulatory, antioxidant, anti-inflammatory, analgesic, and spasmolytic properties [65].

Utilizing acetic acid-induced writhing in mice and hot plate thermal stimulation in rats, it was demonstrated that the essential oils of *Eucalyptus globulus*, *Eucalyptus tereticornis*, and *Eucalyptus citriodora* produced analgesic effects in both models, indicating peripheral and central actions. Additionally, the three Eucalyptus species’ essential oil extracts demonstrated anti-inflammatory properties by inhibiting histamine and carrageenan-induced blood vessel permeability, neutrophil migration into rat peritoneal cavities, and rat paw edema caused by dextran and carrageenan [66].

### 3.7. Thyme Essential Oil

There are numerous members of the Lamiaceae family, *Thymus* genus. These plants, which are native to the Mediterranean region, are frequently used in cosmetics, food preparation, and medicine [67]. The key component of thyme essential oil, thymol, has been showed to have antioxidant, antibacterial, antiviral, antifungal, expectorant, anthelmintic, antiseptic, and antispasmodic properties [68].

Thyme, clove, and tea tree essential oils were found to suppress the *Cutibacterium acnes* and *Staphylococcus epidermidis* growth in a microbiological screening. The thyme essential oil had the most potent and rapid bactericidal effect, eliminating the initial bacterial inoculum with *Cutibacterium acnes* and *Staphylococcys epidermidis* after 10 h and 6 h of exposure, respectively. The highest anti-biofilm activity was also seen with thyme essential oil. Furthermore, it displayed its antibacterial activity by damaging the acne-associated bacteria cell membrane, causing the release of its cytoplasmic components [25].

Three different experimental models, including carrageenan-induced pleurisy, ear edema, and in vitro chemotaxis, were used to examine the effects of *Thymus vulgaris* essential oil and the separated components thymol and carvacrol. *Thymus vulgaris* essential oil, carvacrol, and thymol all had a substantial inhibitory effect on inflammatory edema in the pleurisy model. Only *Thymus vulgaris* essential oil and carvacrol, though, suppressed leukocyte migration. While thymol had a strong chemoattractant impact in the in vitro chemotaxis trial, carvacrol prevented leukocyte migration [69].

An experimental study assessed the bioactive components in four essential oils derived from *Origanum vulgare, Origanum heracleoticum, Thymus serpyllum,* and *Thymus vulgaris*, and evaluated the effectiveness of these essential oils against *Salmonella enteritidis,* based on their antibacterial and anti-biofilm properties. In the past, strains were designated as bdar morphotype (i.e., brown, dry, and rough) or rdar (i.e., red, dry, and rough) based by how the extracellular matrix constituents curli fimbriae and cellulose presented. The results of the experimental study demonstrated that carvacrol and thymol showed a suppression of biofilm development at sub-minimal inhibitory concentration. The strains with distinct morphotypes (rdar and bdar) did not exhibit any statistically significant differences in the effectiveness of essential oils in inhibiting biofilm development [70].

### 3.8. Tea Tree Essential Oil

The value of plants as a source of therapeutic compounds has long been acknowledged. Specifically, secondary plant metabolites like essential oils have been valued for medicinal purposes throughout history. Tea tree oil, commonly known as melaleuca or melaleuca oil, is an essential oil that is steam-distilled out of the Australian native plant *Melaleuca alternifolia* (Myrtaceae family) and has anti-inflammatory and antibacterial properties [71].

There are around 100 terpenes and the corresponding alcohols associated with them in tea tree oil, which has a well-defined composition. Commercial tea tree oil must adhere to an international standard that regulates its physical and chemical characteristics. Tea tree oil exhibits in vitro antiviral, antifungal, antibacterial, and anti-inflammatory activities, indicating potential applications in the management of skin infections. Clinical results have shown that the use of tea tree oil is effective in removing carriers of methicillin-resistant *Staphylococcus aureus*, in addition to the treatment of oral candidiasis and acne [72].

To assess effectiveness and skin tolerance using 5% tea-tree oil gel and 5% benzoyl peroxide lotion in treating mild to moderate acne, a single-blind, randomized clinical experiment was conducted on 124 individuals. According to the study’s findings, both 5% benzoyl peroxide and 5% tea-tree oil significantly improved the acne of the study participants by reducing the amount of non-inflamed and inflamed lesions (i.e., open and closed comedones); however, tea-tree oil took longer to present therapeutic effect. The fact that patients receiving tea-tree oil reported fewer negative effects is promising for future research directions [73].

A randomized, double-blind clinical trial involving 60 patients with mild to moderate acne vulgaris evaluated the efficacy of topical 5% tea tree oil gel compared to a placebo. The trial spanned 45 days, with patients assessed every 15 days. Results indicated that tea tree oil gel demonstrated significant improvement over placebo in terms of total acne lesion count and acne severity index [74].

An open-label, uncontrolled phase II pilot study aimed to assess the efficacy, tolerability, and acceptability of tea tree oil gel and face wash for treating mild to moderate facial acne. A total of 18 participants were enrolled, with 14 completing the 12-week study period. Efficacy was measured based on total facial acne lesion counts and investigator global assessment scores, both demonstrating significant improvement over time. Adverse events were minimal, and local tolerability issues resolved without intervention. The study’s conclusion highlights the significant improvement in acne and favorable tolerability of tea tree oil products [75].

Another clinical study involving 60 patients with mild to moderate acne vulgaris employed a randomized division into three groups. One group received treatment with a cream containing 20% propolis, 3% tea tree oil, and 10% aloe vera (PTAC), while the other groups were administered a 3% erythromycin cream (ERC) or a placebo. Importantly, the study’s findings highlighted the superior effectiveness of the PTAC formulation compared to ERC in reducing erythema scars, acne severity index, and total lesion count [76].

### 3.9. Rosemary Essential Oil

The widely used culinary herb rosemary (*Rosmarinus officinalis* L.) has been shown to have anti-inflammatory, antioxidant, anti-antimicrobial, and anti-carcinogenic properties, along with a number of other health advantages [77]. Numerous investigations have shown that LPS-induced inflammation is inhibited by rosemary extract or its constituents [78].

In a study targeting *Cutibacterium acnes*, rosemary essential oil showed an MIC of 0.56 mg/mL. Atomic force microscopy measurements of *Cutibacterium* acnes revealed significant morphological and size changes in reaction to essential oil therapy. As the concentration of the essential oil increased, the bacterial cells experienced significant damage [79].

Another experimental study assessed the rosemary (*Rosmarinus officinalis*) extract’s ability to reduce *Cutibacterium acnes*-induced inflammation both in vivo and in vitro. The findings revealed that, in *Cutibacterium acnes*-activated monocytic THP-1 cells, ethanolic rosemary extract significantly inhibited the mRNA expression and production of proinflammatory cytokines, like IL-1β, IL-8, and TNF. Simultaneous intradermal injection of ethanolic rosemary extract reduced the granulomatous inflammation and ear edema caused by *Cutibacterium acnes* in an in vivo mouse model [80].

Clinical studies evaluating the potential of essential oils in the management of acne vulgaris are extremely limited in number. ClinicalTrials.gov lists only two such trials, NCT01657110 and NCT04045119; these trials have been completed, but their results have not yet been published. Therefore, it is imperative that in vitro studies be supported by more solid in vivo data through the conduct of more studies [81].

**Figure 3:**
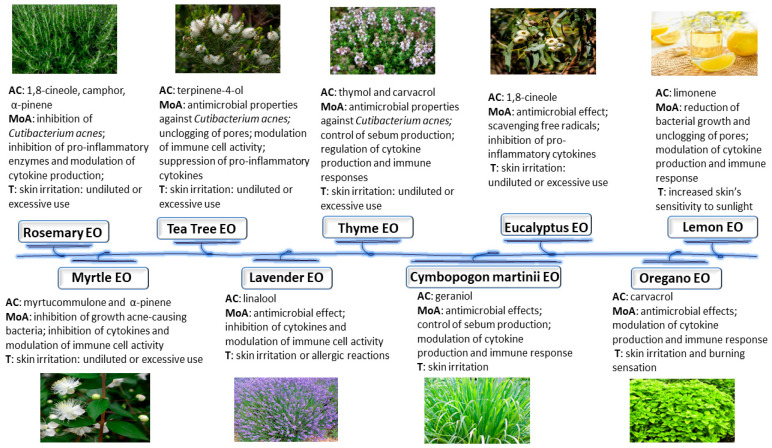
Summary of active components, modes of anti-inflammatory and antibacterial action, and toxicity of essential oils in the field of acne vulgaris. EO, essential oil; AC, active components; MoA, mode of action; T, toxicity.

Figure 3 presents a summary of the active components, modes of anti-inflammatory and antibacterial action for the management of acne vulgaris, and toxicity data of the essential oils presented above [18,26,82].

## 4. Antioxidant Activity of Certain Essential Oils

Specific essential oils’ biological antioxidant processes have an essential role in ensuring and sustaining optimal health and well-being. Antioxidants are compounds that protect cells against the damage triggered by free radicals, which are harmful molecules in specific biologic contexts. Furthermore, free radicals can cause oxidative stress, which has been linked to inflammatory processes, chronic illnesses, aging [83], and the development of acne vulgaris [84].

Certain essential oils contain a wide variety of bioactive molecules, including flavonoids, terpenes, and phenols, making them a good source of antioxidants. These substances have a good capacity to neutralize free radicals while preventing oxidative cell and tissue damage. By scavenging and inhibiting the formation of free radicals, essential oils lower the risk of cellular harm, inflammatory processes, and the onset of chronic diseases [85,86].

### 4.1. Oregano Essential Oil

Oregano essential oil is widely recognized not only for its strong antibacterial properties and unique flavor, but also for its potent antioxidant activity. Several experimental investigations on its biological antioxidant processes and pharmacological properties have been conducted [87,88,89].

Given its high concentration of phenolic compounds, particularly carvacrol and thymol, oregano essential oil possesses potent antioxidant properties [90]. These compounds possess a potent ability to scavenge free radicals and efficiently mitigate oxidative stress in tissues and cells. Although oregano essential oil exhibited high antioxidant activity, it was lower than that of butylated hydroxytoluene. Moreover, leaf-flower oils exhibited the highest antioxidant activity. In the 2,2′-azino-bis(3-ethylbenzothiazoline-6-sulfonic acid) (ABTS) assay, the antioxidant activity of *Origanum compactum* essential oils was comparable to that of Vitamin C [91]. It has been suggested that a potential synergy between oregano essential oil’s constituents might explain their antioxidant potential [92].

When sows received supplements with essential oils of *Origanum vulgare* subsp. *hirtum*, the oxidative stress indicators in their offspring were reduced, as evidenced by reduced blood levels of 8-hydroxydeoxyguanosine and TBARS, which cause severe DNA damage [93]. It has been suggested that, depending on the dose, certain antioxidants can function as both pro-oxidants and antioxidants. In this regard, carvacrol and thymol can trigger oxidative stress in several Caco-2 cells if applied in high amounts, as these terpenes may increase the level of ROS and diminish the GSH content [94,95].

GC-MS was used to determine the chemical components of the three essential oils that were extracted from oregano roots, flowers, stems, and leaves. Applying the reducing power assay and the DPPH test for free radical scavenging, the antioxidant capacity of each essential oil was evaluated. The stem oils are the least effective at serving as antioxidants among the essential oils extracted from various oregano components, while the leaf-flower oils possess the greatest antioxidant properties. The IC50 levels for the essential oils were (0.501 ± 0.029) mg/mL for stems, (0.357 ± 0.031) mg/mL for roots, and (0.332 ± 0.040) mg/mL for leaves/flowers, according to the data gathered from the DPPH free radical scavenging assay [96].

Oregano essential oil has demonstrated effectiveness as an antioxidant and may have the capacity to delay lipidic oxidation. In addition, oregano essential oil inhibited the autoxidation of polyunsaturated fatty acid esters isolated from rodent brains, and carvacrol and thymol were the antioxidative components [97].

Oregano essential oil increased the protein concentrations of the nuclear factor-erythroid 2-related factor-2 target genes, γ-glutamyl cysteine ligase, and Cu/Zn-SOD1, along with the intracellular levels of SOD1 and GSH, in a dose-dependent way. In IPEC-J2 cells, oregano essential oil additionally enhanced intranuclear nuclear factor-erythroid 2-related factor-2 target gene expression and the functioning of an antioxidant response factor reporter plasmid. Moreover, oregano essential oil significantly suppressed ROS and MDA [98].

In the context of acne vulgaris, the antioxidant properties of oregano essential oil may mitigate the oxidative stress-induced damage to skin cells, thereby reducing inflammation and fostering healing. Furthermore, oregano essential oil may contribute to the overall improvement of acne symptoms by neutralizing free radicals [47].

### 4.2. Cymbopogon Martinii Essential Oil

Distinctive in its chemical composition, *Cymbopogon martinii* essential oil has gained scientific interest due to its potential in biological antioxidant mechanisms and pharmacological activities. It is most commonly used to treat skin infections such as acne and to stimulate cell regeneration while regulating sebaceous production [99].

Experimental studies have shown that a variety of bioactive compounds, including geraniol and geranyl acetate, contribute to the essential oil of *Cymbopogon martinii’s* antioxidant properties [52,99,100]. Geranyl acetate and geraniol function as free radical scavengers, protecting cells from oxidative damage and mitigating the effects of oxidative stress [52,99].

Through the reducing power assay, nitrogen oxide assay, DPPH assay, beta-carotene bleaching assay, and FRAP method, an in vitro evaluation of the antioxidant activity of *Cymbopogon martinii* essential oil was conducted. In either the reducing activity or FRAP methods, the reducing activities increased proportionally to the concentrations. The results demonstrate that *Cymbopogon martinii* essential oil is effective at neutralizing free radicals and has the potential to be an effective antioxidant in acne management [99].

### 4.3. Lavender Essential Oil

According to biochemical analysis, several bioactive substances, such as linalool and linalyl acetate, contribute to the antioxidant properties of lavender essential oil. Linalyl acetate and linalool are the two main components of lavender essential oil and have demonstrated antioxidant properties by scavenging free radicals and decreasing oxidative stress [101].

In a cellular model, the antioxidant effects of lavender essential oil have been studied. The study showed that lavender oil protected cells from oxidative injury by boosting antioxidant enzyme activity and decreasing ROS production [102].

The effects of *Lavandula angustifolia* essential oil on the levels of enzymatic and non-enzymatic antioxidants [i.e., SOD, glutathione peroxidase (GPx), and GSH] in in vitro (human hepatoma cell line HepG2) and ex vivo (freshly isolated rat hepatocytes) structures have been measured. In both samples pre-treated with *Lavandula angustifolia* essential oil, the number of oxidant-induced DNA lesions was significantly reduced. Both the increase in GPx activity in cells pre-treated with *Lavandula angustifolia* essential oil and the antioxidant activity of *Lavandula angustifolia* essential oil could account for the observed DNA-protective effect [103].

The antioxidant potential of various essential oils is strongly correlated with their capacity to scavenge radicals and neutralize singlet oxygen, as well as their reducing capacity. In an experimental study, the potential antioxidant capacity of *Lavandula angustifolia* essential oil was measured by its ability to scavenge stable DPPH free radicals. The results revealed that the antioxidant capacity of the essential oils indicated a moderate level of antioxidant activity [104]. In a similar study, the antioxidant activity of essential oils extracted from *Lavandula angustifolia* ranged from 12% (hydro distillation and hexane extraction) to 63% (supercritical CO2-extraction), depending on the extraction technique [105].

A total of 16 components were found in the essential oil of *Lavandula dentata*. Linalool (45.06%) was the most prevalent compound, followed by camphor (15.62%) and borneol (8.28%). Using the DPPH and FRAP testing methods, the essential oil indicated a substantial antioxidant activity, with IC50 and half maximal effective concentration levels of 12.95 ± 1.300 mg/mL and 11.88 ± 0.23 mg/mL, respectively. The total antioxidant capacity of *Lavandula dentata* essential oil was 81.28 ± 2.28 mg ascorbic acid equivalents/g essential oil, with all the assessments showing promising antioxidant potential [106].

Linalyl acetate, linalool, trans-ocimene and geranyl acetate, were found to be the main constituents of the *Lavandula angustifolia* essential oils according to the results of GC (gas chromatography), MS (mass spectrometry) and flame ionization detection. The most potent antioxidant action was demonstrated by Xinxun3 oil; however, all the evaluated essential oils showed considerable antioxidant action. In terms of improved collagen regeneration action, Xinxun2 exhibited the most notable ferrous ion chelating function alongside increased reducing capability [107].

Although there are not many research studies on the use of lavender essential oil in acne vulgaris, the oil’s antimicrobial, anti-inflammatory, and antioxidant properties indicate its potential benefits. Further research is required to investigate its efficacy, optimal formulation, and safety in the treatment of acne.

### 4.4. Myrtle Essential Oil

Analytical examination of myrtle essential oil revealed the presence of numerous bioactive compounds, such as α-pinene, myrtenyl acetate, and 1,8-cineole, that contribute to its antioxidant properties. It has been demonstrated that these compounds possess substantial antioxidant effects via multiple biochemical pathways. Moreover, α-pinene has been shown to neutralize free radicals and inhibit lipid peroxidation, thereby protecting cells from oxidative damage. Inhibiting ROS production and improving antioxidant enzyme activities, 1,8-cineole has also demonstrated promising antioxidant properties [108].

The capacity for scavenging ABTS free radicals by myrtle essential oil obtained from *Myrtus communis* had been greater than the capacity for scavenging DPPH free radicals, given that concentrations of the samples below 1 mg/mL were sufficient to provide 50% inhibition in the ABTS approach, while the maximum scavenging percentage of DPPH was in the range of 41% at 2 mg/mL [109].

Chelating metal ions could avoid lipid peroxidation because transition metal ions may promote lipid peroxidation by boosting the production of initiating species, accelerating peroxidation, and decomposing lipid hydroperoxides that sustain the process of lipid oxidation [110]. There have been fewer studies assessing the antioxidant capacity of myrtle essential oils using cell culture models and methodologies. It has been reported that, when ROS production in a spontaneously immortalized human keratinocyte cell line was triggered by either hydrogen peroxide or UV-B radiation, resulting that the addition of 10% myrtle essential oil reduced production by 40% to 60% [111].

In an experimental study, the impact of myrtle essential oils on rodents with intestinal ischemia/reperfusion-induced oxidative stress was investigated. The effects observed showed a decreased MDA production and an increased GSH, SOD, CAT, and GPx activity [112]. In addition, myrtle essential oil’s antioxidant and anti-inflammatory properties contribute to its potential for reducing inflammation and fostering the healing of acne-prone skin.

### 4.5. Lemon and Other Citrus Essential Oil

Lemon oil and other derived essential oils, such as those obtained from orange, grapefruit, and bergamot, are widely recognized for their potential medicinal uses. These essential oils have been the subject of extensive research regarding their biological antioxidant processes, pharmacological activities, and potential use in the treatment of acne vulgaris [26,113].

Twenty-one components were identified in *Citrus limon* essential oil, with limonene and β-pinene being the most abundant. This *Citrus limon* essential oil exhibited robust β-carotene bleaching inhibition with an IC50 of 40.147 g/mL. This essential oil contains monoterpenes that may function as radical scavengers. The antioxidative characteristics of essential oils containing monoterpene hydrocarbons, sesquiterpenes, and oxygenated monoterpenes appear to exhibit a growing scientific trend of interest and investigation. *Citrus limon* essential oils contain 1,8-cineol, α-pinene, β-pinene, and limonene, which are responsible for these effects [113].

Monoterpene hydrocarbons, particularly terpinolene, γ- and α-terpinene, could also be considered for the reported antioxidative action, but none has a higher antioxidative effect than oxygenated monoterpenes, probably due to the presence of highly activated methylene groups in these molecules. Sesquiterpene hydrocarbons and their oxygenated derivatives, on the other hand, have very low antioxidant activity. The aforementioned findings demonstrated that *Citrus limon* essential oil possesses natural antioxidant properties that are superior to those of butylated hydroxytoluene [113,114].

Limonene, a significant component of *Citrus limon* essential oils, is a potent free radical scavenger that protects cells from oxidative damage and shows antioxidant properties by suppressing lipid peroxidation and decreasing oxidative stress [115].

By applying gas-liquid chromatography, the antioxidant properties of *Citrus limon*, *Citrus paradisi*, *Coriandrum sativum*, and *Caryophyllus aromaticus* essential oils and their mixtures were examined. By applying the oxidation of the aliphatic aldehyde hexanal to the carboxylic acid, antioxidant activity was determined. *Citrus paradisi* and *Caryophyllus aromaticus* essential oils exhibited the lowest and highest antioxidant activities, respectively [116].

In an experimental analysis, lemon essential oils produced through cold pressing as opposed to hydro distillation were compared for non-volatile content and antioxidant properties. According to pathological findings, cold-pressed lemon essential oil was more efficient than hydro distilled lemon essential oil at preventing liver damage induced by oxidative stress. In mice experiencing oxidative damage, cold-pressed lemon essential oil enhanced serum GSH, SOD, and GSH-Px levels while lowering IL-1β, IL-6, nitric oxide, cyclooxygenase-2, and TNF-α levels. Cold-pressed lemon essential oil had effects comparable to vitamin C and more powerful than hydro distilled lemon essential oil effects. Cold-pressed lemon essential oil downregulated the expression of inducible nitric oxide synthase, TNF-α, IL-1β, cyclooxygenase-2, and nuclear factor kappa-light-chain-enhancer of activated B cells mRNA and the expression of nitric oxide synthases and cyclooxygenase-2 protein while upregulating the expression of Cu/Zn-SOD, catalase, and Mn-SOD, in oxidatively damaged mice [117].

Due to their antibacterial, anti-inflammatory, and antioxidant properties, citrus essential oils may provide multiple benefits for acne vulgaris management.

### 4.6. Eucalyptus Essential Oil

The primary component of eucalyptus oil, eucalyptol, possesses potent antioxidant properties by scavenging free radicals and reducing oxidative stress. Inhibiting lipid peroxidation and minimizing oxidative damage, α-pinene, and limonene have also demonstrated antioxidant properties [118].

The neuroprotective effects of different polarity extracts (i.e., ethanol, acetone, and methanol) from *Eucalyptus globulus* leaves were examined in order to identify their key bioactive components. These effects are based on the antioxidant activity of Eucalyptus essential oil. The largest concentrations of phenolic compounds were found in the methanol and acetone extracts, and chlorogenic acid was the main component recognized by ultrahigh-performance liquid chromatography-electrospray ionization-tandem mass spectrometry. The three extracts that were evaluated also shown notable antioxidant activities, with the intensity of these qualities changing according to the in vitro method used. Additionally, *Eucalyptus globulus* extracts proved to be efficient at reducing the generation of ROS and lipid peroxidation rates in SH-SY5Y cells while increasing cell survival, GSH levels, and antioxidant enzyme activity [119].

### 4.7. Thyme Essential Oil

It has been demonstrated that thymol is the major compound in thyme essential oil, and antibacterial, antifungal, antiviral, antihyperglycemic, and antioxidant properties have been observed in it. Carvacrol, terpinen-4-ol, p-cymene, linalool, γ-terpinene, and β-myrcene may further contribute to or modulate these effects [120].

In an experimental study, the activity examinations of the antioxidant enzymes CAT and SOD, in addition to the assessment of the total antioxidant capacity of *Pseudomonas aeruginosa*-activated THP-1 cells, showed that thyme essential oil increased catalase and SOD activity as well as the THP-1 cells’ antioxidant capacity [121].

The chemical components, antioxidant activities, and biological processes of *Thymus quinquecostatus* essential oils were investigated in a study. Carvacrol ethyl ether, a novel natural compound, first appeared in the thyme oils. Additionally, the Kelch-like ECH-associated protein 1/Nuclear factor erythroid 2-related factor 2 pathway contributed to essential oils’ antioxidant processes [122].

### 4.8. Tea Tree Essential Oil

The tea tree essential oil extracted from its leaves contains terpinen-4-ol, terpinolene, 1,8-cineole, α-terpinene, and γ-terpinene as significant constituents. Due to the presence of these bioactive compounds, tea tree essential oil may possess potent antioxidant properties and function as an antioxidant and radical scavenger by preventing the excessive production of ROS [123].

Chemical analysis revealed that α-terpinene degrades rapidly, producing allylic epoxides, p-cymene, and hydrogen peroxide as the primary oxidation products. Consequently, the oxidation pathway differs from that of limonene, which produces highly allergenic hydroperoxides as the main oxidation products upon autooxidation. Moreover, α-terpinene can be considered a true antioxidant because it autoxidizes quickly relative to numerous other compounds, preventing their decomposition [124].

Through dichloro-dihydro-fluorescein diacetate and dihydroethidium probes, it has been demonstrated that tea tree essential oil inhibits the formation of intracellular hydrogen peroxide and superoxide anion radicals in *Caenorhabditis elegans*. It has been determined that tea tree oil’s antibacterial, anti-inflammatory, and antioxidant properties reduce the number of inflammatory lesions, particularly papules and pustules, suggesting its possible use in acne vulgaris management [125].

### 4.9. Rosemary Essential Oil

**Figure 4:**
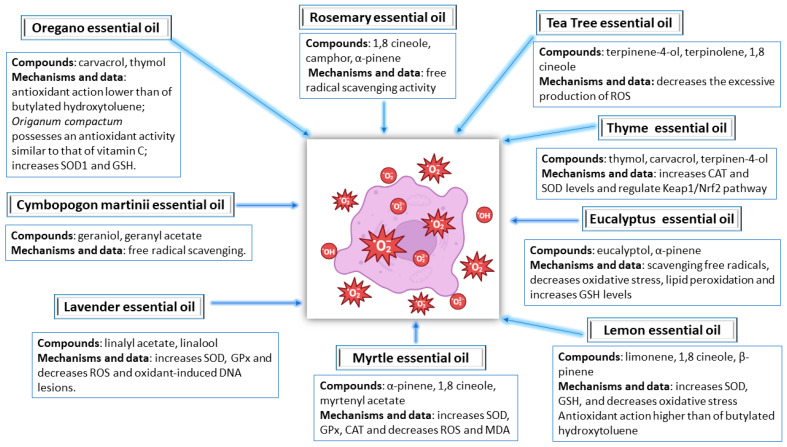
Antioxidant action of certain essential oils. SOD, superoxide dismutase; GSH, glutathione; GPx, glutathione peroxidase; ROS, reactive oxygen species; DNA, deoxyribonucleic acid; CAT, catalase; MDA, malondialdehyde; Keap1/Nrf2, Kelch-like ECH-associated protein 1/Nuclear factor erythroid 2-related factor 2.

Rosemary essential oil has been used in the food industry as a preservative due to its antioxidant and antimicrobial properties, but it has been shown to have additional health benefits. Rosemary essential oil consists mainly of 1,8-cineole, camphor, and α-pinene. The results obtained after antioxidant assays showed substantial free radical scavenging activity and lipid peroxidation inhibition, indicating rosemary oil’s potent antioxidant capacity and a potential use in the management of acne vulgaris [126,127]. Figure 4 shows a summary of the antioxidant effects of the essential oils presented above.

It has been suggested that the antioxidant properties of essential oils are at least partially related to the processes by which they affect oxidative stress. Free radical scavenging activities, inhibition of pro-oxidation, and regulation of antioxidant enzymes (i.e., SOD, catalase, GSH etc.) have been reported as the main antioxidant mechanisms. It has been considered that the longer the conjugated terpenes, the higher the scavenging activity. Furthermore, it has been reported that the compounds of essential oils can be used as antioxidants that can contribute to the prevention of chronic illnesses and neurological conditions that are linked to oxidative stress, including acne vulgaris [93].

## 5. Analysis of Essential Oils

### 5.1. Techniques Employed for the Extraction, Isolation, and Purification of Essential Oils

Extraction, isolation, and purification are key steps in obtaining high-quality essential oils from plant materials. These processes aim to extract the aromatic compounds and other beneficial components while removing impurities. The quality of essential oils exhibits a direct correlation with the chosen extraction method. Elevation of temperature and prolonged extraction durations contribute to quality deterioration. Preserving the chemical composition and inherent proportions of essential oils during extraction is imperative. Noteworthy laboratory-level innovations in extraction encompass: supercritical fluid extraction, subcritical liquid extraction, solvent-free microwave extraction, and pulsed electric field extraction [128].

Given the intricate composition of extracts, additional separation and purification steps are necessary to yield bioactive products. High-performance thin-layer chromatography (HPTLC) and optimum performance laminar chromatography (OPLC) are employed at the laboratory scale. These methods of approaching high performance layers facilitate the separation of natural substances and impurities based on physical or chemical differences within a sample [128,129]. On an industrial scale, essential oils are extracted through processes like hydrodistillation and steam distillation. Furthermore, essential oil isolation and purification entail techniques such as TLC and high-performance liquid chromatography (HPLC) [128,130].

### 5.2. Common Analytical Methods for Determining the Antioxidant Activity of Essential Oils

The scientific community has shown a heightened interest in exploring antioxidants and their implications across diverse disciplines, encompassing important domains such as medicine. This spike in interest can be attributed to the increasing number of publications focused on this subject, signifying a growing recognition of the importance of antioxidants and their potential applications. In the course of the last several decades, the methods and instruments applied for assessing antioxidants’ activity have undergone significant advancements. Earlier methodologies measured the effectiveness of antioxidants against the development of specific species of oxidation products and consequently depended on lipid oxidation measurements. Presently, multiple chemical analyses coupled with highly accurate and automated identification techniques are utilized in assessing antioxidant activity [131].

Chromatography is an effective technique for determining and identifying essential oil components. GC and HPLC are the two types of chromatographic techniques most frequently employed for essential oil analysis. HPLC is utilized for the separation and identification of non-volatile and semi-volatile compounds, whereas GC is utilized for the assessment of volatile compounds. The integration of MS in GC or HPLC methods facilitates the separation of constituents based on their physical and chemical characteristics, followed by ionization and mass spectrometer detection. These methodologies provide invaluable information regarding the chemical composition of essential oils [132,133,134]. Furthermore, there are numerous widely used methods for assessing the antioxidant potential of essential oils: total phenolic content (TPC), DPPH free radical scavenging assay, ABTS assay, FRAP assay, ferrous ion chelating activity, TBARS assay, and antioxidant activity measurement with the β-carotene bleaching (BCB) test [135,136,137,138,139].

An experimental study evaluated the antioxidant activity of *Artemisia dracunculus* essential oil obtained from the plant’s aerial parts. The total concentrations of phenol compounds, proanthocyanidins, and flavonoids, as well as the antioxidant potential in the various extracts, have been evaluated by four distinct antioxidant activity evaluations: DPPH free radical scavenging, ferrous chelating activity, trolox equivalent antioxidant capacity (TEAC), and reducing power assay. The compounds in the essential oil found in the highest proportion are estragole, methyl eugenol, germacrene D, and bicyclogermacrene, and the class of compounds found in the greatest concentration is phenyl propanoeids (67.6%) [140].

The most common method for determining the TPC of essential oils is the Folin–Ciocalteu approach [141], where the Folin–Ciocalteu reagent is responsible for oxidizing the phenolic substances in the item being analyzed. This reagent consists of a combination of phosphotungstic acid and phosphomolybdic acid. After phenols are oxidized, they are reduced to a mixture of blue tungsten and molybdenum oxides. The maximal absorption of the resulting blue color occurs around the wavelength of 750 nm, and it correlates to the total concentration of phenolic substances initially present [142,143,144].

The essential oils generated by the steam distillation process from needles of six China-endemic conifer species (i.e., *Pinus massoniana*, *Pinus henryi*, *Pinus tabulaeformis*, *Pinus tabulaeformis var. mukdensis*, *Pinus tabulaeformis f. shekanensis*, and *Pinus tabulaeformis var. umbraculifera*) were analyzed using GC-MS technique. The assessment of TPC was determined by the Folin–Ciocalteu colorimetric approach using gallic acid as a standard, and the results were displayed in gallic acid equivalents (GAE). Furthermore, TPCs of the herbal extracts varied from 86.60 to 138.34 mg GAE/100 g dry weight, with *Pinus tabulaeformis var. mukdensis* having the highest value [145].

DPPH is a widely utilized, cost-effective, and simple method for measuring antioxidant activity and utilizes free radicals to evaluate the capacity of compounds to function as donor molecules of hydrogen or free-radical scavengers. The free radical DPPH reacts with an odd electron to produce a significant absorbance at the wavelength of 517 nm, which results in a purple color. Upon interaction with the source of hydrogen atoms, the DPPH solution undergoes a transition to a reduced state of diphenyl picrylhydrazine, leading to the disappearance of the purple color [146,147,148]. The outcomes are easily reproducible and equivalent to those of other free radical scavenging techniques [149]. The antioxidant efficacy is determined at room temperature to eliminate the possibility of thermal breakdown of the analyzed compounds [150]. Another commonly used method for the determination of the antioxidant potential relies on the reduction of the ABTS cation radical [151].

The FRAP assay is a simple, automated test that measures the ferric-reducing capacity of various compounds. Under conditions of low pH, the conversion of ferric ions to ferrous ions triggers the formation of a colored complex involving ferrous ions and tripyridyl triazine. Comparing the absorbance variation at 593 nm in test reaction mixtures versus those comprising ferrous ions at an established concentration yields FRAP values. Variations in absorbance are proportional over a broad range of concentrations for antioxidant mixtures. Moreover, the FRAP assay is cost-effective, utilizes a simple and rapid practical protocol without requiring complicated operations based on complex preparation, and has a high degree of precision and reliability in the measurement of antioxidant capacity [152,153].

FRAP is a distinctive test that measures antioxidants (or reductants) in a sample as opposed to other methods which evaluate free radical suppression. The results obtained from the FRAP assay show the amount of electron-donating antioxidants that correspond to the reduction of ferric iron to ferrous ion [154,155].

An experimental study aimed at assessing the chemical composition and antioxidant properties of the essential oil extracted from the Chilean leaves of *Aloysia polystachya* (Griseb) Moldenke, and, via GC-MS analyses, eight compounds were identified, with R-carvone, dihydrocarvone, and R-limonene being the most significant. The antioxidant potential has been assessed by total reactive antioxidant potential (TRAP), DPPH, and FRAP, exhibiting promising antioxidant activity when compared to commercial controls [156].

The TBARS assay is commonly used to quantify lipid oxidation and antioxidant potential. The TBARS assay’s prolonged duration of reaction time might impede its utility as a rapid screening method for antioxidants, potentially affecting its ability to deliver reliable and consistent results. Moreover, the test entails the interaction of lipid peroxidation items, mainly MDA and TBA, which produce MDA-TB2 adducts known as TBARS. TBARS produce a red-pink color that may be spectrophotometrically measured at a wavelength of 532 nm [157,158].

The antioxidative properties of the essential oil from *Alpinia calcarata* Roscoe (Zingiberaceae) rhizomes have been examined. The DPPH free radical scavenging assay and the TBARS assay were carried out to investigate the antioxidant potential. The outcomes of the experimental studies indicated that the essential oil extracted from the rhizomes of *Alpinia calcarata* possesses moderate antioxidant activity [159,160].

The BCB assay measures the antioxidant properties of plant extracts by measuring their capacity to inhibit β-carotene oxidation in a mixture of linoleic acid free radicals. Under these conditions, linoleic acid generates free radicals through the removal of hydrogen atoms from its methylene groups. Furthermore, β-carotene is exceedingly sensitive to these types of free radicals, and its double bonds are oxidized, causing it to lose its color. The degree of β-carotene decolorization indicates the quantity of free radicals in the entire system. The existence of an extract with antioxidant properties inhibits or avoids the initial oxidation of linoleic acid, preserving the yellow/orange hue of β-carotene [161,162,163].

**Figure 5:**
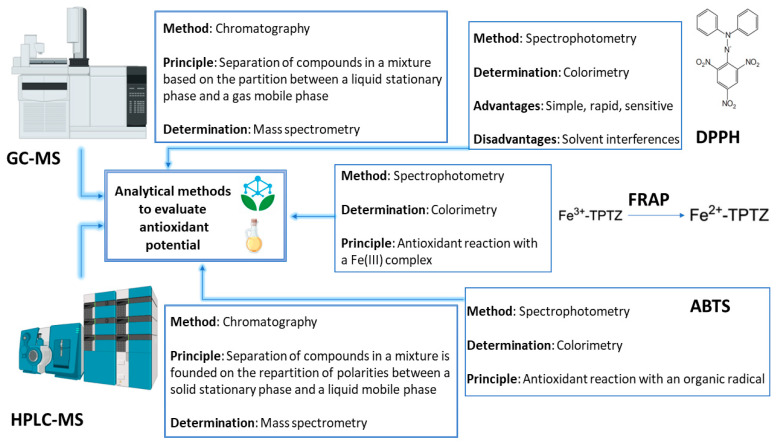
Analytical techniques used prevalently for the determination of the antioxidant potential of volatile oils. GC-MS, Gas Chromatography-Mass Spectrometry; HPLC-MS, High-Performance Liquid Chromatography-Mass Spectrometry; DPPH, 2′-diphenyl 1-picrylhydrazyl; FRAP, ferric-reducing antioxidant power; TPTZ, tripyridyl triazine; ABTS, 2,2′-azinobis(3-ethylbenzothiazoline-6-sulfonic acid).

Figure 5 shows some of the most commonly applied analytical methods for separation, identification, and analysis of the antioxidant potential of essential oils [164].

## 6. Conclusions

The potential application of essential oils as an alternative or complementary treatment for acne vulgaris is promising. Their antioxidant, anti-inflammatory, and antimicrobial benefits render them useful for targeting the numerous factors that contribute to acne vulgaris’ development.

Essential oils are evaluated for their antibacterial and antioxidant effects using various analytical methods. However, essential oils are intricate mixtures of volatile and non-volatile compounds, rendering their analysis difficult. To precisely assess the molecular composition and bioactive properties of essential oils, a well-defined analytical method and an optimized protocol are required.

Although essential oils have demonstrated progress as a treatment for acne vulgaris, several knowledge deficits remain. Future areas of study may focus on the design of new formulations incorporating essential oils, the assessment of their efficacy in clinical trials for the treatment of acne vulgaris, the determination of optimal concentrations and safety profiles, and a better understanding of their underlying mechanisms.

## Data Availability

Data provided in the manuscript are supported by the inserted references.
